# Validation of a MALDI-TOF MS Method for SARS-CoV-2 Detection on the Bruker Biotyper and Nasopharyngeal Swabs: A Brazil—UK Collaborative Study

**DOI:** 10.3390/diagnostics13081470

**Published:** 2023-04-19

**Authors:** Otávio A. Lovison, Raminta Grigaitė, Fabiana C. Z. Volpato, Jason K. Iles, Jon Lacey, Fabiano Barreto, Sai R. Pandiri, Lisiane da Luz R. Balzan, Vlademir V. Cantarelli, Afonso Luis Barth, Ray K. Iles, Andreza F. Martins

**Affiliations:** 1 Laboratório de Pesquisa em Resistência Bacteriana (LABRESIS), Hospital de Clínicas de Porto Alegre, Porto Alegre 90035-903, RS, Brazil; 2 Núcleo de Bioinformática (Bioinformatics Core), Hospital de Clínicas de Porto Alegre, Porto Alegre 90035-903, RS, Brazil; 3 Programa de Pós-Graduação em Ciências Farmacêuticas, Universidade Federal do Rio Grande do Sul, Porto Alegre 90160-093, RS, Brazil; 4 Map Sciences Ltd., The iLab, Priory Park, Bedford MK44 3RZ, UK; 5 Laboratório Federal de Defesa Agropecuária, Porto Alegre 91780-580, RS, Brazil; 6 Grupo Exame, Diagnósticos da América S.A. (DASA), Novo Hamburgo 93310-002, RS, Brazil; 7 Departamento de Ciências Básicas da Saúde, Universidade Federal de Ciências da Saúde de Porto Alegre, Porto Alegre 90050-170, RS, Brazil

**Keywords:** COVID-19, SARS-CoV-2, MALDI-TOF, mass spectrometry, viral screening, diagnostics, population screening, epidemiology, proteomics

## Abstract

We developed a MALDI-TOF mass spectrometry method for the detection of the SARS-CoV-2 virus in saliva-gargle samples using Shimadzu MALDI-TOF mass spectrometers in the UK. This was validated in the USA to CLIA-LDT standards for asymptomatic infection detection remotely via sharing protocols, shipping key reagents, video conferencing, and data exchange. In Brazil, more so than in the UK and USA, there is a need to develop non-PCR-dependent, rapid, and affordable SARS-CoV-2 infection screening tests that also identify variant SARS-CoV-2 and other virus infections. In addition, travel restrictions necessitated remote collaboration with validation on the available clinical MALDI-TOF—the Bruker Biotyper (microflex^®^ LT/SH)—and on nasopharyngeal swab samples, as salivary gargle samples were not available. The Bruker Biotyper was shown to be almost log10^3^ more sensitive at the detection of high molecular weight spike proteins. A protocol for saline swab soaks out was developed, and duplicate swab samples collected in Brazil were analyzed by MALDI-TOF MS. The swab collected sample spectra that varied from that of saliva-gargle in three additional mass peaks in the mass region expected for IgG heavy chains and human serum albumin. A subset of clinical samples with additional high mass, probably spike-related proteins, were also found. Further, spectral data comparisons and analysis, subjected to machine learning algorithms in order to resolve RT-qPCR positive from RT-qPCR negative swab samples, showed 56–62% sensitivity, 87–91% specificity, and a 78% agreement with RT-qPCR scoring for SARS-CoV-2 infection.

## 1. Introduction

Brazil is an upper-middle-income country with 210 million inhabitants in a large territorial area. There is substantial socioeconomic heterogeneity among its five macro-regions, which is reflected in the health services, including the availability of hospital beds and trained healthcare workers [1]. In 2021, the Ministry of Health of Brazil published the “National Plan to Expand Testing for COVID-19” [2]. In order to address the objectives of the plan, clinical and research laboratories have expanded their routines to perform RT-qPCR and high-throughput automated testing that was implemented mainly in reference centers. However, despite the effort made, the laboratories became overloaded while having to deal with SARS-CoV-2 testing kits and laboratory consumables shortages [3].

In addition to insufficient sanitary regulation, inadequate orientation for the population, official communication not based on scientific evidence, people refusing to wear masks, not observing social distancing, and believing in unproven “miracle pills” (widespread self- or prescribed medication with chloroquine/hydroxychloroquine and ivermectin), the pandemic followed an uncontrolled rhythm, reaching 82,869 notified cases and 4.211 deaths in a single day [4]. The intra-hospital mortality was high, and many people died in the absence of ICU beds and respiratory support [1]. Further, by the end of September 2022, Brazil had 34,672,524 cases and 686,036 deaths from COVID-19 [4]. In this scenario with limited resources, the development of rapid and cost-effective screening tests is needed to manage this or new viral pandemics.

The use of MALDI-TOF mass spectrometry in clinical bacteriology has had a substantial impact on the cost of microbiological testing, increasing its availability [5,6]. In this context, this technology could be used for SARS-CoV-2 screening as well as other viruses screening with a lower cost and faster turnaround time, which is extremely important to improving healthcare in Brazil. 

A MALDI-TOF mass spectrometry technique for SARS-CoV-2 detection had been developed by Prof. Ray Iles and his team in the UK, with the potential to detect asymptomatic and pre-symptomatic infections [7,8]. The virus contains a limited number of distinctive proteins that are expressed by hijacking the host cells’ housekeeping and protein synthesis systems. This MALDI-TOF MS method can detect SARS-CoV-2 proteins after enrichment and extraction steps. Since the virus is replicating and, thus, expressing these structural proteins that are targeted by the method, even when the patients are asymptomatic, it is possible to detect the SARS-CoV-2 infection [8]. 

In this context, an international collaboration was proposed between MAP Sciences UK and institutions in southern Brazil, to trial and validate the methodology using the Bruker Biotyper (microflex^®^ LT/SH) and nasopharyngeal swab samples. In order to achieve the aim, the UK team at MAP Sciences established and validated the performance characteristics of Brazilian equipment against the Shimadzu 8020 MALDI-TOF mass spectrometer; and the Brazilian team prepared, collected, and analyzed swab samples for which RT-qPCR results had been obtained. Data analysis was performed by MAP Sciences. 

## 2. Materials and Methods

### 2.1. Samples

A pseudotype lentiviral constructs expressing the SARS-CoV-2 were obtained from Professor Nigel Templeton of the Pseudotype Unit at the University of Essex, UK. In addition, acetone-extracted culture media from Hek293 cells infected with SARS-CoV-2 (the original UK isolation of the Alpha variant) were obtained from Jonathan Heeney of the Laboratory of Viral Zoonotics, Department of Veterinary Medicine, at the University of Cambridge, UK. Seven lentiviral pseudotype constructs were used (SARS-CoV-1, SARS-CoV-2, MERS, NL3, OC43, 229E, and HKU1).

Gargle samples from 222 individuals from Bedford, UK, were obtained from volunteer asymptomatic individuals in Bedford, UK, and collected by MAP Sciences researchers between the 7 July 2020 and the 14 February 2022. In addition, 37 saliva-gargle samples for COVID-19 patients, who had recovered and were 3 months after discharge from the COVID respiratory care wards of Papworth Hospital, were collected by Dr. Helen Baxendale and colleagues of Cambridge Universities Hospital NHS Trust, Cambridge, UK. 

Nasopharyngeal swab samples were collected in May 2021 from Porto Alegre, South Brazil, for the MALDI-TOF validation protocol, and 248 were randomly selected for this study. These samples were previously submitted to RT-qPCR according to CDC protocol. Among them, 100 were considered positive (Ct < 40) and 148 negative (Ct > 40) for SARS-CoV-2 by RT-qPCR. In total, 92.3% (229/248) of the patients reported respiratory symptoms.

### 2.2. MALDI-TOF Mass Spectrometry

In the UK, a Shimadzu 8020 MALDI TOF owned by MAP Sciences and a loaned Bruker Biotyper (microflex^®^ LT/SH, Bruker, Coventry, UK) from Bruker were available for the study. The Brazilian research laboratory at Porto Alegre runs a Bruker Biotyper (microflex^®^ LT/SH), which was used to profile isolated virion envelope proteins. 

The initial development for viral detection by MALDI-TOF mass spectrometry was designed for gargle samples and used a Shimadzu 8020 MALDI-TOF mass spectrometer. Thus, the first experiments were a direct comparison of a Bruker Biotyper on the same pseudotype SARS-CoV-2, extracted and cultured SARS-CoV-2, and the same saliva-gargle samples analyzed by a Shimadzu 8020 MALDI-TOF. This was conducted in the UK at MAP Sciences in the UK [7]. 

The second consideration was adapting the pre-existing sample processing protocols for saliva-gargle samples to utilize the nasopharyngeal swab sample collected in Brazil. Upon the sample collection, swabs were inserted and stored in 1 mL of saline. Thus, we had to work with a limited volume of a sample to empirically identify the most reliable method for viral protein extraction and enrichment prior to mass spectrometry. As opposed to the gargle sample, no filtration was necessary as the large particulate matter was not a major interfering factor. The following method was applied: 500 µL of the soak-out swab saline was mixed with 500 µL ice-cold (4 °C) acetone. The samples were placed in a centrifuge and spun at 16,000 RCF at 4 °C for 30 min. The resulting pellet was reconstituted in 50 uL LBSD-X buffer (MAP Sciences, Bedford, UK) with 20 mM tris(2-carboxyethyl)phosphine (TCEP), and it was plated in duplicates in a sandwich technique after 15 min with a 15 mg/mL concentration of sinapinic acid (SA) matrix. 

Initially, the calibration of the Bruker Biotyper was set using the established and recommended calibrants and fitting curve for biotyping as provided and recommended by Bruker. Subsequently, in the UK only, both the Shimadzu 8020 MALDI-TOF mass spectrometer and the Bruker Biotyper (microflex^®^ LT/SH, Bruker, Coventry, UK) were calibrated using a 2-point calibration of 2 mg/mL bovine serum albumin (33,200 *m*/*z* and 66,400 *m*/*z*). Mass spectral data were generated in a positive ion, linear mode. For the Bruker Biotyper, the laser power was set at 65%, and the spectra were generated at a mass range between 10,000 and 200,000 *m*/*z*; pulsed extraction was set to 1400 ns.

### 2.3. Bioinformatics

Data files were quality-checked, identifying a reference peak in a region of 10,000–11,900 *m*/*z* as previously described [8]. Spectral data were preprocessed by smoothing (a single-cycle, Gaussian smoothing method with a window size of 150 *m*/*z* and a baseline correction). Peak picking as a maximum height was performed with a 1% deviation from the predefined range of key peaks. Peaks that were not consistent throughout the dataset were removed, leaving a total of 24 features. Missing peaks on the spectra were imputed with 0.01.

Based on our previous studies on saliva-gargle samples and antibody analysis, peaks were assigned to immunoglobulin chains and, where present, SARS-CoV-2 proteins [9,10,11]. 

In an undirected peak/mass spectrum comparison approach, machine learning (ML) was applied as follows. Prior to algorithm training, the data were checked for normality, and a 60–40% test train split was made. Decision tree-based algorithms performed best, and the grid search hyperparameter tuning was applied to the Random Forest and Extra Tree Classifiers with the best parameters. Additionally, the out-of-bag score was used on the random forest for confidence in the accuracy of the predictions.

## 3. Results

**Figure 1:**
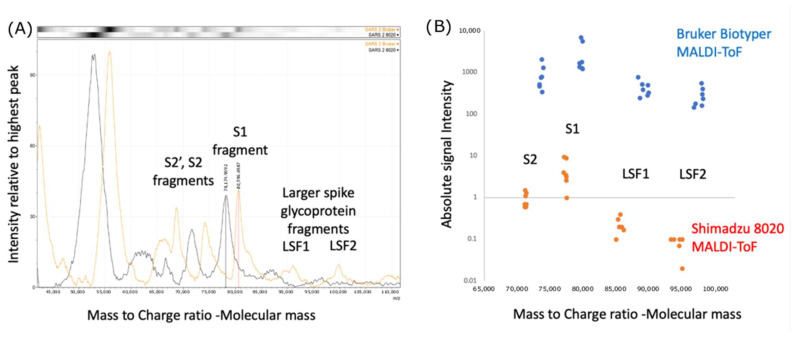
Calibration and Intensity differences Bruker Biotyper versus Shimadzu 8020 mass spectra: (**A**) comparison of peak masses found for Lentiviral pseudotype constructs expressing the Envelope spike protein of the SARS-CoV-2 Spike gene. S1—Spike protein proteolytic fragment 1; S2—Spike protein proteolytic fragment 2; S2′ Spike protein proteolytic fragment 2′; LSF—Large spike glycoprotein fragments; Bruker Biotyper—red line spectra; Shimadzu 8020—black line spectra; (**B**) comparison of mass and intensity difference for seven LentiViral pseudotype constructs expressing the Corona Virus’ respective Envelope Spike genes (SARS-CoV-1, SARS-CoV-2, MERS, NL3, OC43, 229E, HKU1). Blue dots—Bruker Biotyper; Orange dots—Shimadzu 802.0.

Comparing the performance of the Bruker Biotyper with the Shimadzu 8020 MALDI-TOF on pseudotype lentivirus expressing the SARS spike protein complex, we found that the peak masses were consistently higher by 3–4% in mass, and the intensity of the higher mass peaks was also higher in recorded intensity by log10^3^ (see Figure 1).

**Figure 2:**
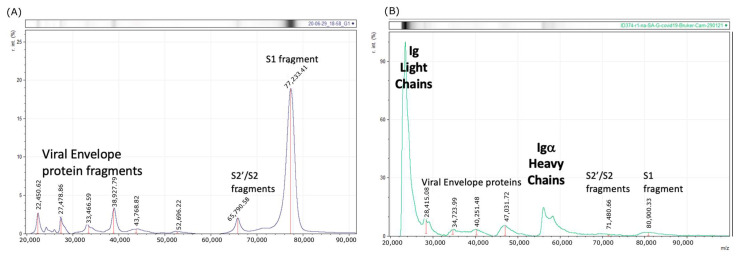
Bruker Biotyper Mass spectral profiles after higher mass drift correction by two-point (H^+^ and 2H^+^) calibrating with BSA: (**A**) In Vitro spectra—Culture supernatant, UK isolate of COVID-19 (first wave alpha) grown on HEK293 cells now match with Shimadzu 8020 spectra (Figure 1A—black line). (**B**) Clinical spectra—second wave saliva-gargle from asymptomatic RT-qPCR positive—Male 60’s. S1—Spike protein proteolytic fragment 1; S2—Spike protein proteolytic fragment 2; S2′ Spike protein proteolytic fragment 2′.

Additionally, by recalibrating the Bruker using bovine serum albumin (BSA) as a two-point calibrant (single and double charged BSA), the masses on subsequent spectral analysis of in vitro cultured SARS-CoV-2 (alpha) matched (see Figure 2). 

**Figure 3:**
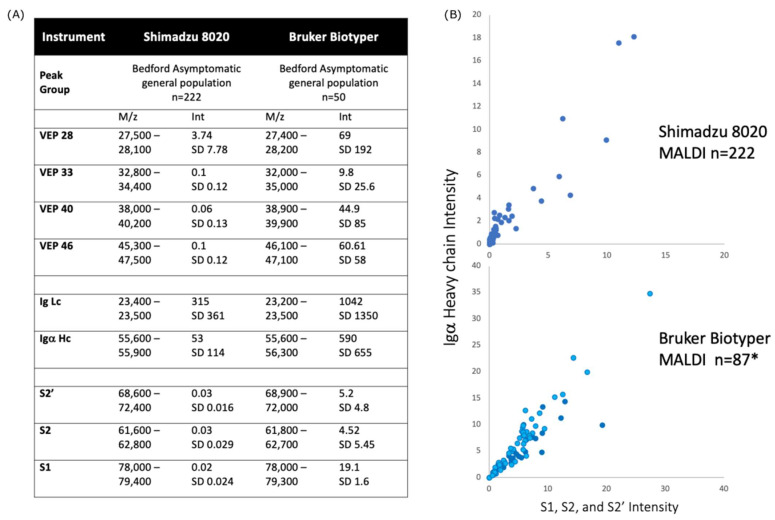
Comparison of spectral data generated from the Shimadzu 8020 and Bruker Biotyper MALDI-TOF mass spectrometer on saliva-gargle samples (**A**) tabulation of mass and intensity of viral envelope, immunoglobulin and spike fragments. VEP—Viral envelope protein. The annotation number was based on the *m*/*z* of the peak; Lc—Light chain; Hc—Heavy chain; S1—Spike protein fragment 1; S2—Spike protein fragment 2; S2′—Spike protein fragment 2′; *m*/*z*—mass to charge ratio; Int—peak intensity; SD—standard deviation; (**B**) plot of Igα heavy chain intensity versus spike protein (Spike protein fragments S1, S2 and S2′) intensity for the Shimadzu 8020 (upper panel) and Bruker Biotyper mass spectrometers. There is a strong correlation between Igα heavy chain and spike protein fragments intensity. * refers to an additional 37 convalescent COVID-19 patient gargle samples added to the plot (dark blue circles).

In looking at saliva-gargle samples, where calibration had been corrected to the method employed in the development of the Shimadzu 8020 SARS-CoV-2 assay; the Bruker Biotyper produced spectra with matching peaks to those generated by the Shimadzu 8020 MALDI-TOF MS. However, these were between 50 and 100 times higher in intensity on the Bruker Biotyper MALDI spectra than the same saliva-gargle samples run on the Shimadzu 8020 MALDI-TOF MS (see Figure 3). Consequently, a higher cutoff value would be needed to distinguish between positive and negative samples. 

Furthermore, among 248 Brazilian swab samples, 215 produced spectra that met QC criteria (86.7%). Of these, 79 were SARS-CoV-2 RT-qPCR positive and 136 were RT-qPCR negative. These spectra revealed slightly different patterns. First of all, note that samples run in Brazil had a mass shift, which can be attributed to the machine calibration employed for bacterial biotype not precisely matching high-mass protein mass fitting. At high masses, the line fitting dependent average mass shift was a 700–4000 *m*/*z* higher value than those recorded in the UK values for immunoglobulin chains (Ig light chains approx. 23,000 *m*/*z*) and SARS-CoV-2 viral envelope proteins (highest detected being approx. 100,000 *m*/*z*). This was not corrected in silico, as such manipulation may have introduced a bias in the ML interpretation of the results.

**Figure 4:**
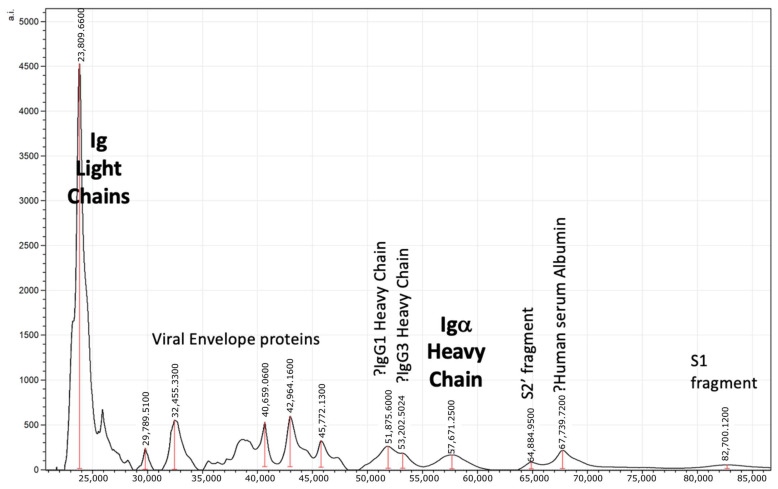
Example spectra of swab sample from SARS-CoV-2 RT-qPCR Brazilian patient with peak identification annotation after allowing for a 3–4% drift in higher mass calibration. S1—Spike protein fragment 1; S2—Spike protein fragment 2.

Significantly, the presence of three new peaks corresponding to the expected masses of human serum albumin (HSA) and Igγ1 and Igγ3 heavy chains was a common finding in nasopharyngeal swab samples and very rarely seen in gargle samples. This was in addition to Ig light chains and Igα heavy chains, which were the dominant immunoglobulins found in saliva-gargle samples (Figure 4). In a subset of nasopharyngeal swab samples, additional higher mass peaks greater than 80,000 *m*/*z* could also be seen.

**Figure 5:**
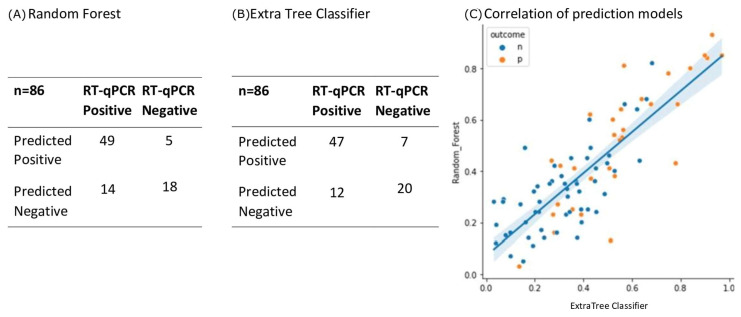
Performance characteristics of ML algorithms. Of the 215 sample spectra, 129 (60%) were randomly selected to form the training set. It was found that Random Forest (**A**) and Extra Tree Classifier (**B**) ML algorithms performed the best. A testing set of 86 samples (which had been independent of the training set) were run on the two algorithms and the results shown in the two confusion matrices (Panel (**A**,**B**) respectively). Agreement with RT-qPCR classification was 78%, *p* < 0.00001 for both models. There was a strong correlation between both models on sample scoring for these validation test samples (panel (**C**), line represents linear correlation r^2^ =0.681 and shading the 95% confidence limits. Blue dots (*n*) = negative, orange dots (*p*) = positive.

Given the drift in mass, changes in mass, and new peaks being found, ML was used to detect any spectral pattern correlating with RT-qPCR positive swab samples. The ML system incorporated all 23 spectral features (peak presence and intensity), and the agreement with RT-qPCR was 78% for both random forest and extra tree algorithms, and a strong correlation exists between both ML methods (Figure 5). 

## 4. Discussion

The policy of RT-qPCR for SARS-CoV-2 testing varies among countries depending on the nature of the healthcare system. Within the UK and USA, community and asymptomatic surveillance testing programs encouraged testing individuals who did not present with symptoms. Consequently, throughout the pandemic, 30–40% of positive RT-qPCR results for SARS-CoV-2 were reported in individuals without symptoms [12]. This supported the argument that RT-qPCR testing could detect pre-symptomatic and asymptomatic infections and therefore help to control the spread of the disease [13]. However, the definition of asymptomatic being RT-qPCR positive with no other clinical confirmation of infection, or subsequent development of symptoms, does become a self-serving metric; and does not account for any false positive finding in RT-qPCR testing [13]. 

We used a RT-qPCR with specific nucleocapsid (N) primers N1 and N2 as recommended by the CDC [14], and we found that, among the Brazilian clinical samples analyzed, 92.3% of the patients with positive results for the RT-qPCR reported respiratory symptoms. Although in assessment studies no false-positive results were found when using these primers against in vitro cultures of other respiratory viruses; the majority (53%) of nasopharyngeal samples evaluated in this study presented RT-qPCR with Ct > 35, which indicates low viral load. While in leverage/sputum samples from the same patients, 72% were RT-qPCR positive with Ct < 35 [14,15].

The clinical laboratory protocol in Brazil was to re-test all samples with a Ct between 35 and 39 using CDC N1 and N2 primers (indeterminate results) with the Seegene: E, RdRP, and S and N primer reagent kits [14]. The latter simultaneously detects three target genes specific for SARS-CoV-2: RNA-dependent RNA polymerase (RdRP), N specific for SARS-CoV-2, and envelope (E) for all sarbecovirus (including SARS-CoV-2). However, only rarely were all three target genes detected, as 65% of COVID-19 nasopharyngeal samples presented amplification of one or two of the genes [15]. Thus, two of the three SARS-CoV-2 genes amplified (Ct < 35) were regarded as being positive [15]. Therefore, re-evaluation of indeterminate results with Seegene was considered a strategy to improve detection rates without compromising specificity. Nevertheless, for the samples in the study, this approach was not required, since all positive samples presented a Ct < 35 and negative samples presented a Ct > 40. However, the self-serving definition of asymptomatic as being RT-qPCR positive without ever developing symptoms requires a complementary but orthogonal technology to RT-qPCR testing for SARS-CoV-2 infection detection.

Indeed, due to the costs of RT-qPCR testing (Table 1) and its low availability outside large urban centers, the lateral flow devices that immunologically detect viral antigens have been introduced in many countries. However, this method presented lower sensitivity and only confirmed infection in those individuals with symptomatic disease. In fact, the detection of viral antigen by lateral flow technique presented extremely poor correlation with RT-qPCR pre-symptomatic and asymptomatic detection [13,16,17]. In this context, mass spectrometry was proposed as a more sensitive and affordable method of antigen detection, and our group built a system based on MALDI-TOF MS analysis for viral proteins [8], which was validated by other countries and on different clinical samples. 

The MALDI-TOF MS generally requires very low volumes of generic reagents, and the major cost is the price of the mass spectrometer equipment. The costs of the reagents used in the analysis are around 1.00 USD per sample. Considering other consumable costs and paying the cost of a mass spectrometer over 3 years in operation (at 100,000 samples a year per machine), the retail price would reach approximately 10.00 USD per sample. Despite the reduced costs, the sensitivity is lower compared with molecular methods. An overview of the current available methods and their performance indicators is demonstrated in Table 1.

The issues with travel restrictions during the pandemic have meant that emerging new technology from one country could only be replicated in another through written and verbal communication and not via demonstration and in-person pedagogy. This had been the case in the previously reported validation of our MALDI-TOF mass spectral analysis technique for SARS-CoV-2 infection based on saliva-gargle samples by a USA CLIA laboratory. This had specifically evaluated SARS-CoV-2 viral infection in those individuals without symptoms [8]. For the Brazilian validation reported here, this was further complicated by not having identical MALDI-TOF mass spectrometers and by different clinical samples (saliva-gargle versus nasopharyngeal swabs).

The prevailing sampling method for upper respiratory viruses is nasal–pharyngeal swabs. A few methods have claimed to detect different mass spectra patterns that correlate with SARS-CoV-2 infection from the direct application of swab samples onto MALDI-TOF plates, followed by alpha-Cyano-4-hydroxycinnamic acid (CHCA) matrix addition and spectra acquisition in the 2–20,000 *m*/*z* mass range [18,19,20,21]. Recently, a study with an analogous approach reported similar results from saliva samples [22]. Despite the complex bioinformatics workflow and the robustness of the results, these studies were poorly controlled. Since the comparison of the MALDI-TOF method was made with RT-qPCR-positive versus RT-qPCR-negative samples only, it is difficult to establish whether these spectral patterns are specific for SARS-CoV-2 infection since other respiratory infections were not evaluated. The method reported here addresses the specificity issue through sample enrichment and a targeted interpretation approach that aims for SARS-CoV-2 antigen detection.

The UK results showed extremely comparable data but greater sensitivity with respect to large viral glycoprotein detection by the Bruker Biotyper (Figure 1, Figure 2 and Figure 3). However, the initial calibration settings of the Bruker Biotyper instrument were a matter of concern. Basically, the process and regression equation fit used in the Biotyper MALDI-TOF MS in Brazil for bacterial identification, as set by the manufacturer for bacterial ribosomal proteins, caused a drift of molecular mass overestimation of the much higher masses of viral glycoproteins. Although in the UK all equipment settings were allowed to be changed by the user, in Brazil, as their instrument was routinely used for bacterial identification, this was not allowed to be changed by the user to develop viral studies such as this one reported here. 

The processing of swabs rather than saliva-gargle samples presented only small changes in the overall preparation and was just a matter of volume adjustments. However, it has to be noted that a peak mass consistent with human serum albumin was more prominent, as were two peaks consistent with the masses seen in other studies for Igγ1 and Igγ3 heavy chains [9]. This may be due to the fact that there is an increased exudate due to the physical mucosal tissue abrasion of swabbing, which is not present in saliva, which is obtained by a gentle wash of a gargle. 

Noteworthy, in a small subset of the Brazilian swab samples, broad peaks at around 90 K and 100 K were also detected and were similar in mass to those found in lenti-viral pseudotypes expressing coronavirus spike genes mass spectra (Figure 1).

Given the increased sensitivity of the Bruker Biotyper and the subtle changes in peak composition (and mass positions), a ML approach to differential analysis was adopted. For both of the ML algorithm systems adopted, there was a 78% concordance with RT-qPCR scoring of infection.

Given that positive RT-qPCR results may not be considered fully definitive of SARS-CoV-2 infection, the results of the MALDI-TOF strongly suggest that this technique can be considered an orthogonal but complimentary screening test for SARS-CoV-2 infection. This is particularly valuable in pre-symptomatic and asymptomatic screening in comparison to lateral flow testing, which presents positive results for no more than 4% of pre-symptomatic/asymptomatic samples identified as positive by RT-qPCR [19]. In this study, 56–62% of RT-qPCR-positive samples were also scored as positive by MALDI-TOF mass spectral profiling, and the samples with negative RT-qPCR results presented an agreement of 87–91% with MALDI-TOF.

Nasopharyngeal swabs are considered the standard sample for diagnostic testing for SARS-CoV-2 [23]. However, this type of sampling method requires professional collection, exposes healthcare workers to biological risk, and is uncomfortable, limiting the patient’s compliance, especially if they are asymptomatic. Due to geographical and methodological limitations, it was not possible to perform a head-to-head study to evaluate the true impact of the variation in specimen collection, processing protocols, and population (pediatric versus adult, early versus later disease course). 

Since this method has been proposed for the mass screening of asymptomatic patients, the high proportion of symptomatic patients in the Brazilian dataset is another limitation of this study.

**Table 1:** Comparison between the cost and performance of SARS-CoV-2 detection methods available at retail in Brazil.

Methods	Sensitivity (%)	Specificity (%)	Estimated Costs *	References
MALDI-TOF MS	56–62	87–91	10.00	[7]; Current study
Lateral flow devices	30.2–98.3 (lower on asymptomatics)	88.9–100	20.00	[24]
Molecular methods (e.g., RT-qPCR, LAMP)	84.8–100	98.9–100	30.00	[25,26]

* Estimated costs per sample (USD) varied substantially through the pandemic and were estimated based on Brazilian current retail prices.

## 5. Conclusions

The method presented in this study is an alternative test for SARS-CoV-2 detection, especially in low- and middle-income countries. This method could provide LMICs with not only a more affordable diagnostic but also an expanded screening capacity. Moreover, in countries where science is neglected, with low investments and people living under constant pressure and discredit, international collaboration, as demonstrated here, is the key to achieving clear and promising results.

As the use of alternative specimens for SARS-CoV-2 detection is needed, studies with saliva are important to demonstrate that it is possible to implement different specimen collections for the same method with minor adjustments. Despite the fact that swabs are still considered the highest-yield sample for respiratory virus diagnostic testing, more studies are necessary to elucidate if this remains true for MALDI-TOF MS methods.

MALDI-TOF mass spectral analysis can be considered a complementary and orthogonal clinical test to RT-qPCR. Where agreement on SARS-CoV-2 infection is found on both tests, in patients presenting without symptoms, the diagnosis of infection as asymptomatic can be confidently made. Where the test results diverge, further analysis of clinical relevance is required as the potential to detect the emergence of variants is a strong possibility.

## Data Availability

The data presented in this study are available on request from the corresponding author. The data are not publicly available because they are being used for further investigation and developments.
